# Novelties and limitations of tissue-engineered materials in treating traumatic nerve injuries: a mini review 

**DOI:** 10.3389/fcell.2025.1603678

**Published:** 2025-09-11

**Authors:** Stefanie Deininger, Andreas Knoll, Nadja Grübel, Andrej Pala, Ralph König, Christian Rainer Wirtz, Maria Teresa Pedro

**Affiliations:** Department of Neurosurgery, Bezirkskrankenhaus Günzburg, University of Ulm, Günzburg, Germany

**Keywords:** nerve guide conduit (NGC), traumatic nerve injury, bioengeneering, tissue engineering, neuroma prevention, translational barriers, bioactive materials

## Abstract

Peripheral nerve injuries remain challenging due to the limited regenerative capacity over long distances and the complexity of repair mechanisms. While autologous nerve grafts are the clinical gold standard, their use is restricted by donor-site morbidity and tissue availability. Tissue-engineered materials such as nerve guidance conduits (NGCs), hydrogels, and bioactive scaffolds offer alternative solutions by providing structural support and delivering trophic, immunomodulatory, or electrical cues. This mini-review categorizes these materials by their functional properties, including drug delivery, cell integration, and electroactivity, and critically assesses their preclinical performance and translational limitations. Natural materials such as collagen and chitosan exhibit good biocompatibility but limited mechanical stability and variability. Synthetic polymers and electroactive materials allow for customization and controlled stimulation but often provoke immune responses or degrade into harmful byproducts. Advanced drug-delivery systems using hydrogels and microspheres enable targeted factor release, yet reproducibility and kinetics remain critical barriers. Cell-integrated constructs, including Schwann cell-like cells and engineered neural tissue, offer high regenerative potential but face challenges in scalability, regulatory classification, and manufacturing. Importantly, many preclinical studies do not benchmark against autografts or address neuroma formation, fibrosis, and delayed regeneration—key issues in human lesions. A summary of preclinical constructs and translational barriers is provided to highlight recurring obstacles such as immune incompatibility, insufficient vascular integration, and regulatory hurdles. Future research must refine model systems, align regulatory strategies, and enhance construct functionality to enable effective clinical translation.

## 1 Introduction

Peripheral nerve regeneration is a complex biological process involving Wallerian degeneration, axonal sprouting, and remyelination. Following injury, Wallerian degeneration is triggered by calcium influx and activation of proteases such as calpain, leading to the systematic breakdown of axons and myelin distal to the lesion (Donnelly and Popovich, 2008; Gaudet et al., 2011). This process creates the foundation for subsequent regenerative events. Schwann cells are pivotal in orchestrating peripheral nerve regeneration. Upon injury, they transition from a myelinating phenotype into a repair phenotype (Bosch-Queralt et al., 2023; Jessen and Mirsky, 2019). These repair Schwann cells actively clear myelin debris, release cytokines and neurotrophic factors, and recruit macrophages, establishing a growth-permissive microenvironment. Importantly, they form Büngner bands–longitudinal cellular columns that guide regenerating axons toward their targets (Jessen and Mirsky, 2019). In contrast to the central nervous system (CNS), where inflammation often impedes repair, the peripheral environment supports regeneration, largely due to Schwann cell plasticity and alignment (Bosch-Queralt et al., 2023). However, successful regeneration across long distances remains limited. Human Schwann cells show reduced metabolic adaptability compared to murine cells, impairing their ability to sustain the energy-demanding repair phenotype and adequately support regeneration (Meyer zu Reckendorf et al., 2020). This contributes to delayed axonal growth and incomplete functional recovery in humans.

Further complications arise from traumatic neuroma formation, which disrupts tissue architecture and promotes chronic inflammation (Deininger et al., 2024; Dömer et al., 2018; Mahan et al., 2019; Karsy et al., 2018; Yang et al., 2023). Neuromas are characterized by disorganized axonal sprouting, persistent inflammatory activation, and excessive angiogenesis, ultimately resulting in a fibrotic and growth-inhibiting microenvironment (Huang et al., 2023). On a cellular level, regenerating axons form aberrant growth cones that fail to establish functional distal connections and show sustained expression of GAP-43, a marker of pathological axon growth, accompanied by infiltration of macrophages, activation and proliferation of fibroblasts and Schwann cells, and the release of pro-inflammatory cytokines such as IL-6, CCL2, and CXCL10, which contribute to sustained inflammation and matrix remodeling (Deininger et al., 2024). In particular, the dysregulated angiogenic response contributes to pathological remodeling by promoting abnormal neovascularization and enhancing immune cell recruitment. Excessive or disorganized microvessel formation can facilitate fibrotic encapsulation. The resulting tissue is structurally disorganized and exhibits aberrant collagen deposition and neoneurovascular crosstalk. These pathophysiological barriers highlight the need for therapeutic strategies that not only support axonal regeneration but also guide outgrowth, modulate inflammation, and suppress maladaptive remodeling, including vascularization and fibrosis.

Autologous nerve grafting remains the clinical gold standard due to its biological compatibility and guidance properties. However, it is associated with donor-site morbidity, limited tissue availability, and inconsistent outcomes in extensive or delayed lesions. To address these limitations, various tissue-engineered materials—such as nerve guide conduits (NGCs), biomimetic scaffolds, and hydrogel-based systems—have been developed to support and direct nerve regeneration (Kehoe et al., 2012; Lam and Leung, 2024). Despite encouraging experimental results, their clinical translation remains restricted due to challenges in standardization, regulatory approval, and long-term efficacy.

This mini-review provides a critical and translationally oriented overview of tissue-engineered materials for peripheral nerve repair. Recent *in vivo* studies, simulating clinically relevant conditions—are categorized by functional strategies such as structural support, drug delivery, bioelectrical modulation, and cell integration. This enables a cross-comparative assessment of design principles and mechanistic rationale. Beyond summarizing clinically approved conduits, we address key translational barriers including regulatory constraints, limited long-term data, and biological complications such as neuroma formation. By linking functional innovation with clinical relevance, this review aims to inform future material development and therapeutic application.

## 2 Materials and their functional applications in nerve repair

### 2.1 Origin of materials

#### 2.1.1 Natural materials

An ideal NGC combines biodegradability, biocompatibility, and sufficient mechanical strength while supporting axonal growth and minimizing inflammation. Natural materials such as collagen, chitosan, and silk have been extensively investigated for their use in NGCs due to their inherent biocompatibility and ability to mimic the extracellular matrix. Collagen, the predominant extracellular matrix protein in nerves, provides a suitable scaffold for cell adhesion and growth. Studies show that collagen-based NGCs effectively support axonal regeneration (Houshyar et al., 2019). FDA-approved collagen-based devices such as NeuraWrap™, NeuraGen®, and Avance® nerve graft have shown promising outcomes in animal studies (di Summa et al., 2014; Mathot et al., 2020). For example, hydrogel-stabilized NeuraWrap™ conduits demonstrate significant axonal outgrowth in preclinical models, although clinical outcomes still lag behind autologous grafts (Georgiou et al., 2013), while NeuraGen® filled with a bioactive hydrogel (AGRG) led to histological and functional outcomes similar to autografts in a chronic 25 mm gap model in rabbits (Rochkind et al., 2021). In a long-gap rat model, NeuraGen® 3D filled with Schwann cells enabled regeneration comparable to autografts (Burks et al., 2021). Moreover, Avance®, a decellularized human allograft, has been successfully seeded with mesenchymal stem cells *ex vivo*, demonstrating high viability and uniform cell distribution (Mathot et al., 2020).

Chitosan, derived from chitin, offers antibacterial properties and tunable degradation rates, making it a promising NGC material. Chitosan and collagen-based conduits facilitated superior functional recovery (Yang et al., 2022) compared to silicone-based conduits due to their bioactive surface properties (Choi et al., 2018). Additionally, silk fibroin, with its robust mechanical properties and ability to support Schwann cell proliferation, has been used to create flexible yet durable conduits. Silk conduits seeded with Schwann cells have shown enhanced axonal alignment and regeneration in rat sciatic nerve models (Das et al., 2015).

Hybrid approaches have also gained attention, combining natural materials to optimize their properties. For example, cellulose/soy protein isolate (SPI)-based conduits demonstrated improved porosity and water absorption, supporting nerve regeneration more effectively than cellulose-only conduits. The sponge-like variant of this hybrid material promoted enhanced axonal growth, though autologous grafts still outperformed these designs in functional recovery metrics (Gan et al., 2016).

Despite these advancements, natural materials’ primary limitations lie in their variability and potential immunogenicity. Future research should explore advanced fabrication techniques and bioengineering strategies to address these limitations by incorporating growth factors or enhancing structural stability (e.g., through cross-linking).

#### 2.1.2 Synthetic materials

Synthetic NGCs leverage advanced materials to replicate the extracellular matrix and support axonal growth. Polycaprolactone (PCL), a widely used biodegradable polymer, is known for its mechanical strength and tunability. Conduits composed of PCL nanofibers have shown improved axonal regeneration compared to microfibers (Jiang et al., 2012). Another promising design involves bilayered scaffolds with random outer and aligned inner nanofibers, which, when seeded with Schwann cells, enhance muscle and nerve regeneration while maintaining structural integrity (Xie et al., 2014).

Electrically active synthetic polymers, such as polylactic-co-glycolic acid (PLGA), further enhance NGC capabilities. Applying electrical fields to PLGA conduits increases neurite outgrowth and nerve conduction, although functional recovery metrics (e.g., muscle strength and sensory function) still lag behind autologous grafts (Bryan et al., 2004). Electrospun PLGA and polycaprolactone scaffolds offer additional flexibility as they can be infused with anti-inflammatory agents or growth factors, thereby enhancing their therapeutic potential (Dziemidowicz et al., 2023).

Recent innovations focus on multi-material approaches to optimize performance. For instance, composite scaffolds made of poly(3-hydroxybutyrate) (PHB) and electrospun nanofibers demonstrated success in bridging long nerve gaps, albeit with slower recovery compared to autografts (Young et al., 2002). Additionally, conductive carbon-based materials such as carbon nanotubes (CNTs) and graphene are being integrated into synthetic conduits to mimic the electrical properties of the nervous system and further promote axonal regeneration under electrical stimulation (Kunisaki et al., 2021). Despite these advancements, synthetic NGCs face challenges related to biocompatibility and immune response. Ongoing research focuses on optimizing material properties (e.g., through surface modification) and developing hybrid designs to improve preclinical efficacy and facilitate clinical translation.

#### 2.1.3 Composite materials

Composite NGCs integrate natural and synthetic components, combining biocompatibility with mechanical strength and functional versatility. Collagen/polycaprolactone (PCL) scaffolds have demonstrated effective Schwann cell adhesion and axonal regeneration comparable to autografts in rat models, emphasizing their potential as a reliable alternative (Yu et al., 2011). Further advancements include poly(L-lactide-co-ε-caprolactone) (PLCL) conduits paired with collagen/hyaluronan hydrogels, which enhanced sensory recovery, although motor function restoration requires further refinement (Jin et al., 2013).

Electrospun composite conduits like poly (lactic-co-glycolic acid)/poly(ε-caprolactone) (PLGA/PCL) have enabled efficient muscle reinnervation and axonal regeneration (Panseri et al., 2008), while intra-luminal channels with aligned nanofibers supported axon alignment and sensory recovery. Notably, these designs sometimes surpassed autografts in certain parameters but often lagged in axon density (Koh et al., 2010).

The incorporation of conductive polymers has further broadened the applicability of composite NGCs. For instance, polypyrrole/chitosan scaffolds under electrical stimulation facilitated improved sensory and motor function, although they did not fully replicate the results of autografts (Huang et al., 2012). Recent studies also highlight using polylactic acid (PLA) fibers combined with neurotrophin-enriched hydrogels to support Schwann cell migration and axonal regrowth, showing promising outcomes in preclinical settings (Quigley et al., 2013).

Overall, composite NGCs offer customizable platforms that address specific challenges in peripheral nerve repair, such as long-gap injuries. However, further optimization in material composition and functional enhancements, such as incorporating growth factors or bioactive molecules, is essential to achieve clinical translation. While these approaches show promise, further refinement is needed to match the efficacy of autologous grafts.

### 2.2 Functional properties of materials

#### 2.2.1 Drug-releasing scaffolds

Enhancing peripheral nerve regeneration through pharmacological support has led to the integration of various therapeutic agents into nerve repair constructs. Among the most investigated substances is the immunomodulatory drug FK506 (tacrolimus), which enhances axonal regeneration and functional recovery. FK506-eluting conduits demonstrated improved regeneration and reduced scarring in rodent models, although autografts remained superior for muscle reinnervation (Azapagic et al., 2024). Similar effects were observed when FK506 or glial cell-derived neurotrophic factor (GDNF) was incorporated into polytetrafluorethylen (PTFE) conduits, resulting in enhanced myelination and functional muscle recovery (Labroo et al., 2019; Kim et al., 2022).

In addition to immunomodulators, growth factor-loaded scaffolds such as those incorporating nerve growth factor (NGF) or brain-derived neurotrophic factor (BDNF) have demonstrated accelerated axonal regrowth and improved outcomes (Zeng et al., 2021; Sandoval-Castellanos et al., 2021). Dual-release systems, for example combining GDNF and BDNF, yielded superior results compared to single-factor strategies (Tajdaran et al., 2019; Dong et al., 2023). In addition to their drug-loading capacity, several commercially approved collagen-based conduits such as Avance® and NeuraGen® offer favorable structural and biological properties for cell seeding, making them particularly suitable platforms for combining scaffold-based and cell-based regeneration strategies (Mathot et al., 2020; di Summa et al., 2014; Burks et al., 2021). In such multifunctional constructs, the interplay between cellular components and bioactive molecule delivery becomes critical. When bioactive agents such as neurotrophic factors or immunomodulators are applied to these scaffolds, their therapeutic efficacy depends not only on the biological activity of the agents themselves but also on the material’s ability to deliver them effectively. Specifically, local concentration, release kinetics, and bioactivity retention are highly dependent on the delivery system used, highlighting the importance of material selection and formulation in nerve repair strategies.

Hydrogels are widely used as therapeutic carrier systems due to their hydrated and tissue-compliant nature. ECM-derived hydrogels, such as those from bone or decellularized nerve matrix, have shown comparable regenerative potential to collagen-based systems (Kellaway et al., 2023; Yang et al., 2022). Synthetic hydrogels allow for tunable viscoelasticity and porosity, supporting cell infiltration, structural integration, and controlled trophic factor delivery (Muangsanit et al., 2021; Saldin et al., 2017). The precision of hydrogel-based systems is further improved by advanced fabrication techniques such as 3D bioprinting.

Recent studies have further underscored the potential of hydrogel-based matrices in peripheral nerve repair. A hydrogel derived from porcine decellularized nerve tissue was shown to support Schwann cell viability and promote functional axonal regeneration in a rat model (Lin et al., 2018). Decellularized optic nerve ECM, while not gelled in the classical sense, retained key bioactive proteins and reduced axonal growth inhibitors, thereby enhancing neurite outgrowth *in vitro* (Sun et al., 2020). In a computational study comparing different delivery strategies, hydrogel films were identified as particularly effective in achieving uniform growth factor distribution within multichannel nerve conduits (Zhou and Vijayavenkataraman, 2022).

Microspheres allow spatially controlled and sustained release of therapeutic agents and are often integrated into composite scaffolds. In nerve injury models, microspheres loaded with FK506 significantly enhanced both motor and sensory axon regeneration (Tajdaran et al., 2019). Chitosan-based microspheres cross-linked with tripolyphosphate (TPP) preserved NGF bioactivity and supported muscle integrity and nerve regrowth (Zeng et al., 2021). Likewise, PLGA-based microspheres delivering anti-inflammatory agents reduced local inflammation and fostered a regenerative microenvironment (Li et al., 2023). Advanced strategies include co-encapsulation of neurotrophic and immunomodulatory agents. For instance, NGF and tacrolimus co-loaded microspheres outperformed single-drug systems in promoting axon density and functional recovery (Nawrotek et al., 2022). Integration into hydrogel or scaffold matrices further enhances localization and retention at the repair site.

Nevertheless, technical challenges remain, including controlling release kinetics, achieving uniform particle sizes, and ensuring biodegradability and safety. Innovations such as nanoparticle coatings and optimized formulations aim to address these limitations (Zhou and Vijayavenkataraman, 2022).

#### 2.2.2 Cell-integrating constructs

Schwann cell-based constructs represent a biologically grounded approach to peripheral nerve repair, leveraging the supportive and regenerative functions of native Schwann cells as essential mediators of peripheral nerve regeneration due to their myelinating and trophic capabilities. When incorporated into biomimetic constructs, they provide directional cues and molecular support for axonal regrowth. For example, collagen hydrogels containing Schwann cells promoted axonal alignment and functional improvement in sciatic nerve models, underscoring the importance of matrix anisotropy (Georgiou et al., 2013). A recent study by Zhu et al. (2025) expanded this concept by co-seeding Schwann cells and sensory neurons onto a decellularized optic nerve scaffold to create a bioactive “tissueoid”. This construct enabled substantial recovery of both sensory and motor function in a rat sciatic nerve defect model, emphasizing the potential of combining native cellular support with preserved extracellular matrix architecture.

Electrospun scaffolds composed of polycaprolactone (PCL) and chitosan enhanced axonal regeneration and cellular adhesion when pre-seeded with Schwann cells (Xie et al., 2014; Wang et al., 2017).

Recent advances also have explored co-culture systems. Combinations of Schwann cells with fibroblasts or endothelial cells have demonstrated increased secretion of neurotrophic factors and ECM components, leading to improved remyelination and axonal outgrowth (Wang et al., 2017). However, limitations in scalability and Schwann cell availability drive interest in alternative cell sources.

Human pluripotent stem cells (hPSCs) can be differentiated into Schwann cell precursors (SCPs) by modulating key developmental pathways such as neuregulin-1/ErbB, Notch, and Wnt signaling (Kim et al., 2017; Chambers et al., 2009). These protocols recapitulate the sequential stages of neural crest development and glial lineage specification. The resulting Schwann-like cells support axonal regeneration and myelination *in vitro* and in preclinical models (Kim et al., 2017). Their scalability and adaptability render them promising candidates for future autologous or allogeneic cell-based therapies.

Recent studies further demonstrate that hPSC-derived Schwann cells maintain functional stability under stress conditions—such as glucotoxic environments—and exhibit disease-relevant responses, highlighting their potential not only in regenerative medicine but also in mechanistic and pharmacological research (Majd et al., 2023).

Among clinically evaluated cell products, the CTX0E03 cell line stands out as a promising candidate. The CTX0E03 cell line is a conditionally immortalized human neural progenitor derived from fetal cortex tissue (Pollock et al., 2006). Originally developed for stroke therapy, this cell line has shown a favorable safety profile and functional benefits in Phase I/II clinical trials (Kalladka et al., 2016; Muir et al., 2020). In models of peripheral nerve injury, CTX0E03 has promoted axonal regeneration and target reinnervation. Its neural origin, clinical-grade manufacturing, and prior use in humans position it as a promising candidate for translation, although its application in peripheral nerve repair remains limited to preclinical studies.

To bypass the lengthy differentiation protocols associated with hPSC-based approaches, direct reprogramming strategies have been developed. These methods enable rapid conversion of somatic cells, such as fibroblasts, into Schwann cell-like cells (SCLCs) without transitioning through a pluripotent or progenitor stage. Bone marrow stromal cells (BMSCs) and adipose-derived stem cells (ADSCs), when stimulated with neurotrophic factors, can also acquire Schwann-like properties and support regeneration comparable to native Schwann cells (Dezawa et al., 2001; Mimura et al., 2004; Kang et al., 2019). ADSC-derived SCLCs have shown particularly promising outcomes in rodent models (Orbay et al., 2012; Tomita et al., 2012).

This strategy has yielded encouraging results in preclinical studies, including improved myelination and functional recovery (Sowa et al., 2017; Wang et al., 2011). Building on this concept, engineered neural tissue (EngNT) constructs have incorporated SCLCs or neural stem cells into aligned, biomimetic scaffolds to further enhance regeneration. These constructs have demonstrated improved vascularization, axonal alignment, and muscle reinnervation *in vivo* (Sanen et al., 2017; O'Rourke et al., 2018; Rayner et al., 2021).

Despite this progress, several translational challenges persist. Differentiation protocols remain time-intensive and variable in efficiency, often requiring several weeks under tightly controlled conditions. Ensuring phenotypic stability and functional integrity after transplantation is critical, as residual undifferentiated cells or partially specified intermediates may compromise safety and efficacy. The immunogenicity of allogeneic hPSC-derived products and the necessity for Good Manufacturing Practice (GMP)-compliant production add further complexity. In Europe, such therapies are classified as Advanced Therapy Medicinal Products (ATMPs), requiring centralized regulatory approval and comprehensive safety evaluation. Nevertheless, advancements in xeno-free differentiation protocols and scalable manufacturing are steadily advancing the clinical viability of hPSC-derived Schwann cells.

#### 2.2.3 Electroactive materials

Electroactive materials, including both conductive and piezoelectric systems, aim to replicate or enhance the electrical environment essential for peripheral nerve repair. By integrating electrical cues into biomaterials, these systems promote axonal growth, Schwann cell activity, and functional regeneration.

Conductive materials utilize externally applied or intrinsic conductivity to stimulate neural tissues. Nanomaterials such as gold nanoparticles (AuNPs), carbon nanotubes (CNTs), and graphene have been incorporated into scaffolds to enhance conductivity and cellular compatibility (Hazer Rosberg et al., 2021; Das et al., 2015; Kunisaki et al., 2021). Silk-based conduits infused with AuNPs improved Schwann cell adhesion and axonal growth over extended periods (Das et al., 2015). Likewise, CNT yarns and reduced graphene oxide (RGO)-coated nanofibers significantly enhanced nerve conduction and cellular alignment under stimulation (Kunisaki et al., 2021; Wang et al., 2019).

Organic conductive polymers, including polypyrrole (PPY) and poly(3,4-ethylenedioxythiophen) (PEDOT), integrate electrical functionality with mechanical stability. PPY/poly(D,L-lactic acid) (PDLLA) scaffolds promoted neurite outgrowth, while PEDOT-coated grafts improved nerve conduction, although their regenerative efficacy remains inferior to autografts (Xu et al., 2014; Baghmanli et al., 2013). These materials highlight the potential of conductive nerve guide conduits (NGCs), though further optimization of biocompatibility and functional outcomes is required.

Piezoelectric materials, in contrast, generate endogenous electrical fields in response to mechanical stimuli. This self-activating property eliminates the need for external stimulation devices. Composites of polycaprolactone (PCL) with zinc oxide have shown accelerated nerve regeneration in preclinical models (Mao et al., 2022). Piezoelectric polymers like polyvinylidene fluoride (PVDF) mimic the electrical behavior of biological tissues and support both axonal regrowth and angiogenesis, especially when combined with external stimulation (Yamazaki et al., 2017). Similarly, black phosphorus-based electroactive scaffolds supported axonal growth and angiogenesis, addressing two key regenerative requirements simultaneously (Qian et al., 2019).

Further developments involve combining piezoelectric scaffolds with bioactive agents. For example, ibuprofen-loaded wraps made of PLA and PCL reduced inflammation while enhancing neurotrophic factor expression and axonal recovery (Dziemidowicz et al., 2023). Aligned nanofibrous scaffolds further improve cellular orientation and tissue integration (Zhang et al., 2020).

Conductive materials provide robust, controllable stimulation via external sources but often require additional devices and may induce higher energy demands. Piezoelectric scaffolds offer autonomous, movement-driven stimulation but may produce lower absolute current outputs. Both strategies present promising solutions yet face distinct translational challenges - ranging from biocompatibility and electrical tuning to long-term safety and degradation control.

### 2.3 Summary of translational barriers of preclinical constructs

Despite extensive progress in the development of nerve guidance conduits (NGCs), the clinical translation of preclinically successful constructs remains limited. While several natural and synthetic materials have advanced to regulatory approval for short-gap sensory nerve injuries (e.g., collagen, chitosan, polycaprolactone-based devices), their utility is restricted by gap length, degradation behavior, and insufficient trophic support (Burks et al., 2021; Böcker et al., 2022; Costa Serrão de Araújo et al., 2017).

A major barrier across nearly all material classes is the mismatch between the mechanical or biochemical properties of the scaffold and the regenerative microenvironment *in vivo*. Synthetic materials, though tunable and reproducible, often degrade into acidic byproducts or show limited cellular integration (Young et al., 2002; Bryan et al., 2004). Natural scaffolds may perform better in terms of biocompatibility but suffer from variability in source material, immunogenic risk, and insufficient long-term stability (di Summa et al., 2014; Choi et al., 2018). These findings are systematically summarized in Table 1, which contrasts representative material strategies by their preclinical outcomes, translational stage, and associated barriers.

**TABLE 1:** Summary of preclinical constructs for peripheral nerve repair, including material type, regenerative outcomes, translational stage, and key barriers to clinical application.

Material Type/strategy	Preclinical outcomes	Translational status	Key barriers	Representative references
Collagen-based NGCs	Support axonal guidance, Schwann cell infiltration, and moderate functional recovery in rat models	Clinically approved (NeuraGen®, Avance®; short- gap sensory repair)	Limited effectiveness >3 cm; enzymatic degradation; batch variability; limited trophic support	Georgiou et al. (2013), di Summa et al. (2014), Burks et al. (2021)
Chitosan-based NGCs	Enhanced axonal regeneration, modulated immune response, and functional recovery in rodents	Clinically approved (e.g. Reaxon® for digital sensory nerves ≤40 mm)	Limited indication scope; poor motor nerve data; degradation rate variability	Yang et al. (2022), Das et al. (2015); Choi et al. (2018), Böcker et al. (2022)
Synthetic NGCs (e.g., PCL, PLGA, PHB, CNTs)	Support axonal regeneration and myelination in rodent/rabbit models; aligned fibers, conductivity, or drug loading enhance efficacy	Clinically approved (e.g. Neurolac® for sensory nerves ≤ 30 mm)	Mechanical mismatch; stiffness; hydrophobicity; limited motor nerve outcomes	Jiang et al. (2012), Bryan et al. (2004), Young et al. (2002), Kunisaki et al. (2021), Costa Serrão de Araújo et al. (2017)
Composite NGCs (e.g., Silk/PCL, PPy/chitosan, PLGA-hydrogel blends)	Enhanced axonal regeneration, Schwann cell migration, and vascularization; effects amplified via electrical stimulation or neurotrophic gels	Preclinical (small animal)	Complex fabrication; material compatibility; degradation control; combination product regulation	Jin et al. (2013), Koh et al. (2010), Panseri et al. (2008), Quigley et al. (2013)
Drug-releasing NGCs (e.g., neurotrophin- loaded scaffolds, ECM hydrogels, FK506 depots, MSC-enhanced conduits)	Improved axonal regeneration, immune modulation, and functional recovery in rodent and large- animal models; enhanced by sustained release of NGF, GDNF, IL-10, FK506	Preclinical (includes large- animal model – canine)	Release kinetics; biologic stability; scalability; classification as ATMPs or combination products	Dong et al. (2023), Mathot et al. (2020), Labroo et al. (2018), Tajdaran et al. (2019), di Summa et al. (2014)
Drug-releasing scaffolds using micro- and nano- carriers (e.g., PLGA microspheres, hydrogel gradients)	Spatiotemporally controlled factor release; improved targeting, Schwann cell behavior, and pain modulation	Preclinical (small animal)	Fabrication complexity; degradation; regulatory burden; limited in vivo longevity	Zhou and Vijayavenkataraman (2022), Nawrotek et al. (2022), Zeng et al. (2021), Li et al. (2023)
Primary Schwann cells in hydrogels, ECM scaffolds or electrospun conduits	Promote axonal alignment and myelination; improved function in rodent models	Preclinical (small animal)	Donor availability; viability; phenotype loss; scalability	Georgiou et al. (2013), Xie et al. (2014), Wang et al. (2017), Zhu et al. (2025)
hPSC- and MSC- derived Schwann-like cells	Support axonal regeneration and myelination; stress-resilient; scalable production	Preclinical (small animal)	Lengthy protocols; GMP adaptation; ATMP classification	Kim et al. (2017), Majd et al. (2023), Kang et al. (2019)
Engineered Neural Tissue (EngNT) with SCLCs or neural stem cells	Functional recovery in long- gap models; autograft-level performance in some studies	Preclinical (includes long- gap models)	Construct complexity; GMP assembly; regulatory coordination	Sanen et al. (2017), O'Rourke et al. (2018), Rayner et al. (2021)
Electroactive scaffolds (e.g., PPy, GO, CNTs, PVDF, PEDOT, PLLA-SPI)	Electrical stimulation enhanced Schwann cell behavior, axonal elongation, and functional recovery; PLLA- SPI matched autograft outcomes (Zhang et al., 2020)	Preclinical (small animal)	Biosafety of nanomaterials; conductivity stability; device integration; regulatory complexity	Yamazaki et al. (2017), Qian et al. (2019), Dziemidowicz et al. (2023), Zhang et al. (2020)

Abbreviations: ATMP, advanced therapy medicinal product; CNT, carbon nanotube; ECM, extracellular matrix; EngNT, engineered neural tissue; FK506, tacrolimus; GDNF, glial cell-derived neurotrophic factor; GMP, good manufacturing practice; GO, graphene oxide; hPSC, human pluripotent stem cell; IL-10, interleukin-10; MSC, mesenchymal stem cell; NGC, nerve guidance conduit; NGF, nerve growth factor; PCL, polycaprolactone; PEDOT, poly(3,4-ethylenedioxythiophene); PHB, polyhydroxybutyrate; PLGA, poly(lactic-co-glycolic acid); PLL, poly-L-lysine; PLLA-SPI, poly(L- lactic acid)–silk protein isolate; PPy, polypyrrole; PVDF, polyvinylidene fluoride; SCLC, Schwann cell- like cell; SPI, silk protein isolate.

Drug-delivering scaffolds represent a particularly promising approach to enhance regeneration through the local release of neurotrophic or immunomodulatory agents. However, challenges in controlling release kinetics, maintaining protein bioactivity, and ensuring reproducibility of delivery systems limit clinical readiness (Labroo et al., 2019; Dong et al., 2023; Nawrotek et al., 2022). Similar constraints apply to scaffolds incorporating micro- and nano-carriers, where precise spatiotemporal control of factor gradients *in vivo* remains difficult to achieve at scale (Zhou and Vijayavenkataraman, 2022; Li et al., 2023).

Cell-integrating constructs, particularly those using primary Schwann cells, stem cell-derived Schwann-like cells, or engineered neural tissues (EngNT), offer high biological relevance and regenerative potential. Nevertheless, they face substantial translational barriers such as phenotypic instability, complex production workflows, and regulatory hurdles associated with advanced therapy medicinal products (ATMPs) (Kim et al., 2017; Majd et al., 2023; Sanen et al., 2017; O’Rourke et al., 2018). In particular, scalable and GMP-compliant manufacturing remains a core obstacle for these cell-based technologies.

Electroactive scaffolds have shown potential in modulating axonal outgrowth and Schwann cell behavior through conductive or piezoelectric properties (Zhang et al., 2020; Qian et al., 2019). Yet, biosafety concerns, insufficient standardization of electrical stimulation protocols, and lack of long-term *in vivo* data currently prevent their clinical deployment.

Importantly, many studies still rely on small animal models with short nerve gaps, which do not sufficiently replicate the clinical scenario of large-gap or mixed nerve injuries. Moreover, a substantial number of preclinical investigations fail to include autologous nerve grafts as a benchmark, despite their role as the current clinical gold standard. This limits the interpretability and translational relevance of regenerative outcomes reported for novel constructs.

In sum, the gap between preclinical innovation and clinical implementation is defined less by the lack of regenerative efficacy *per se* and more by the complex interplay of biological, technical, and regulatory demands. Systematic efforts to standardize preclinical models, include appropriate clinical comparators, scale up manufacturing, and define early translational endpoints are essential to unlock the therapeutic potential of next-generation nerve repair materials.

## 3 Clinical applications and prospects

Preclinical studies show significant promise for NGCs with innovations such as 3D-printed scaffolds and biochemical gradients improving axonal regeneration (Johnson et al., 2015; Bell and Haycock, 2012; Kehoe et al., 2012). Various materials and strategies for peripheral nerve repair have been developed, including synthetic scaffolds, electrical stimulation, drug delivery systems, and cell-based therapies. However, most remain at the preclinical stage, with limited translation into clinical practice. To date, only structure-based nerve guide conduits (NGCs) without pharmacological or cellular components have received regulatory approval for clinical use, reflecting challenges in standardization, regulatory pathways, and long-term safety (Lam and Leung, 2024).

### 3.1 Clinically applied NGCs and their limitations

Clinically approved nerve guide conduits (NGCs) are primarily indicated for short-gap sensory nerve lesions. Collagen-based conduits such as NeuraGen®, polyglycolic acid-based Neurotube®, and CE-marked chitosan-based Reaxon® have achieved meaningful recovery rates of up to 73%–75% for nerve gaps ≤40 mm (Meek and Coert, 2013; Lohmeyer et al., 2009; Böcker et al., 2022). However, evidence for their application in motor or mixed nerves remains limited, and complications such as conduit extrusion and fibrotic encapsulation have been reported (Rinker and Liau, 2011; Chiriac et al., 2012). Clinical studies of synthetic conduits like Neurolac® have shown good outcomes in digital nerve repairs (<25 mm), though paresthesias were frequently reported (Costa Serrão de Araújo et al., 2017). In a multicenter prospective trial, Bertleff et al. (2005) observed sensory recovery comparable to autografts. Bozkurt et al. (2017) reported favorable outcomes in 10 of 11 patients treated with Reaxon®, whereas Chiriac et al. (2012) highlighted a high complication rate in motor and mixed nerves, particularly related to stiffness and integration. Collagen-based conduits were further supported by Bushnell et al. (2008), Wangensteen and Kalliainen (2010), and Lohmeyer et al. (2009), who reported 67%–75% of patients achieving protective sensation and two-point discrimination in gaps <30 mm. Nevertheless, up to 17% required revision surgery due to incomplete regeneration or conduit failure. Processed nerve allografts such as Avance® represent an alternative. The RANGER® study (Safa et al., 2020) involving 385 patients and 624 repairs demonstrated meaningful recovery in 82% of cases - including 71% of mixed and 83% of motor nerves in gaps ≤70 mm. While eliminating donor-site morbidity, these grafts require further evaluation in randomized controlled trials. This need is underscored by a recent meta-analysis, which found no significant difference in meaningful recovery rates between autografts and allografts across sensory and motor nerves and various gap lengths but demonstrated consistently lower outcomes for conduits and higher complication rates (Lans et al., 2023). In this context, a prospective, multicentre controlled clinical trial evaluated the use of Shenqiao^TM^ human acellular nerve graft (hANG) in 72 patients with digital nerve defects ranging from 1 to 5 cm. Compared to direct suture (n = 81), hANGs achieved comparable sensory outcomes, with 65% of patients demonstrating good or excellent recovery in static two-point discrimination at 6 months. No product-related adverse events or immunological reactions were reported, supporting the safety and clinical viability of hANGs for bridging short-to-intermediate sensory nerve gaps while avoiding donor-site morbidity (He et al., 2015). A US-based cost-effectiveness model concluded that Avance® allografts may offer a clinically equivalent yet more cost-efficient alternative to autografts, particularly by avoiding donor-site morbidity and associated long-term costs (Ansaripour et al., 2024).

Recent meta-analyses, including those by Saeki et al. (2018) and Thomson et al. (2022), consistently emphasize the limited quality and generalizability of clinical evidence for nerve guidance conduits (NGCs). While some synthetic and biological devices demonstrated acceptable outcomes in short-gap injuries, most of the underlying studies were retrospective, underpowered, and exhibited substantial methodological heterogeneity. Notably, Thomson et al. included 36 trials but identified only five randomized controlled studies, most of which had short follow-up periods and lacked standardized outcome reporting. This heterogeneity, combined with inconsistent definitions of functional recovery, severely limits the interpretability and pooled effect estimates of the meta-analysis.

Importantly, the authors concluded that no NGC demonstrated superiority over autologous nerve grafting in terms of safety or functional recovery with high-certainty evidence, thereby reaffirming the autograft as the clinical gold standard. These limitations underscore the need for standardized endpoints, longer-term studies, and higher-level trial designs in future conduit research.

This concern is further illustrated by a recent randomized multicenter study evaluating the Reaxon® conduit, which reported inferior long-term outcomes after more than 5 years, including persistent pain, incomplete sensory recovery, and evidence of foreign body reactions due to insufficient biodegradation (Aman et al., 2025). Such findings reinforce the need for longitudinal assessment beyond early-phase success, particularly for biodegradable implants.

### 3.2 Translational landscape and barriers

Although numerous preclinical studies demonstrate regenerative potential of engineered NGCs, clinical translation is often hindered by oversimplified rodent models, lack of standardized outcome measures, and inconsistent comparison to autografts. Many studies focus on short-gap sciatic injuries in young animals with high intrinsic regenerative capacity - scenarios not reflective of complex human injuries prone to fibrosis, neuroma, or delayed repair (Jeyaraman et al., 2024). Regulatory uncertainty and manufacturing barriers further limit progress, especially for bioactive, cell-loaded, or drug-eluting constructs. Only acellular NGCs with passive structural function have reached widespread clinical use to date (Lam and Leung, 2024).

Natural and decellularized materials such as collagen, chitosan, and silk offer high biocompatibility and bioactivity, with clinically applied examples including NeuraGen®, Neuromaix®, and Reaxon®. Chitosan-based Reaxon® conduits have shown minimal short-term complications in sensory digital nerves, but inferior outcomes after 5 years (Aman et al., 2025). Decellularized nerve allografts like Avance® enable bridging of gaps up to 70 mm while avoiding donor-site morbidity (Buncke, 2022). However, variability in raw material, batch consistency, and residual immunogenicity present translational hurdles (Hussein et al., 2024). The development of Avance® required decades of optimization and ultimately underwent reclassification discussions due to increased regulatory scrutiny (Kasper et al., 2020). Most approved applications are limited to short-gap sensory repairs; robust clinical evidence in motor or long-gap injuries remains sparse (Gao et al., 2023).

Synthetic nerve conduits made of synthetic polymers such as poly(glycolic acid) (PGA), polycaprolactone (PCL), and poly(lactide-co-caprolactone) offer mechanical strength, tunability, and scalable production. FDA- and CE-approved examples include Neurotube® and Neurolac® (Buncke, 2022; Guo et al., 2022). Regulatory approval requires ISO 10993 compliance, a standardized framework for the biological evaluation of medical devices, providing guidelines to assess biocompatibility, toxicity, and local tissue responses prior to clinical application and validated degradation and sterility data.

Despite controlled manufacture and consistent properties, synthetic conduits lack intrinsic bioactivity and may release acidic byproducts that impair regeneration (Azapagic et al., 2024). Newer designs, such as 3D-printed thermoplastic polyurethane (TPU) conduits, have shown favorable results in critical-size nerve defects in rats while meeting ISO 10993–6 biocompatibility criteria (Zennifer et al., 2024), but clinical validation is pending.

Combination products and drug-Loaded constructs are Advanced Therapy Medicinal Products (ATMPs) are highly regulated biological therapies, including cell-based, gene, and tissue-engineered products, designed to repair or replace damaged tissues. Multifunctional scaffolds integrating drugs (e.g., tacrolimus, GDNF) or growth factors show enhanced preclinical regeneration but face dual regulatory burdens. These systems are often classified as combination products or, in Europe, as ATMPs, requiring complex validation and manufacturing protocols (Guo et al., 2022; Failli et al., 2025). Neurotrophins like NGF and BDNF lack clinical approval, whereas tacrolimus, already FDA-approved, has shown promise in PLLA-PCL conduits (Azapagic et al., 2024). Design complexity is further illustrated by computational models simulating drug release. Zhou and Vijayavenkataraman. (2022) showed that efficiency and distribution of GDNF depend strongly on scaffold architecture, reinforcing the importance of integrating pharmacokinetics into biomaterial design.

Cell-based therapies and Schwann cell substitutes are limited in clinical use by availability, donor-site morbidity, and phenotype instability. Alternatives such as MSCs, ASCs, NSCs, and iPSC-derived Schwann-like cells (SCLCs) are being evaluated (Wei et al., 2024; Rahimi Darehbagh et al., 2024). These cells exhibit neurotrophic and immunomodulatory effects and can be integrated into scaffolds to enhance regeneration (Xu et al., 2024). However, challenges such as cell sourcing, expansion, phenotypic drift, immunogenicity, and integration into host tissue persist (Wang et al., 2024). Regulatory classification as ATMPs adds further barriers - requiring Good Manufacturing Practice (GMP) - certified production, batch validation, and long-term follow-up (Bellino et al., 2023). To date, no cell-based nerve repair therapy has achieved routine clinical use.

ATMP classification and regulatory divergences demand varying strategies depending on regional frameworks. In the European Union, ATMPs - comprising cell-based and tissue-engineered constructs - are centrally regulated by the EMA and require GMP-compliant production along with robust preclinical and clinical data (Bellino et al., 2023). In contrast, the FDA applies a more flexible classification under Section 351 (biologics) or as combination products, based on the primary mode of action (Guo et al., 2022). The same scaffold may be considered a medical device in the US but an ATMP in the EU if it includes viable cells or is substantially manipulated. Developers must therefore align product design with region-specific regulatory frameworks.

Future directions to improve translation must adopt models that reflect the chronic, fibrotic, and neuroma-prone environment of human injuries. Preclinical benchmarks should include motor function, neuroma suppression, and long-gap repair. Comparative trials against autografts remain essential.

Multifunctional constructs integrating structural guidance, trophic support, and immunomodulation hold promise, but require rigorous validation in translational models. Advances in biofabrication, microenvironment engineering, and regulatory strategy alignment will be crucial to bridge the gap between laboratory success and clinical application.
